# The Affective Meaning of Words is Constrained by the Conceptual Meaning

**DOI:** 10.1007/s10936-019-09663-w

**Published:** 2019-08-13

**Authors:** Zhiguo Hu, Hongyan Liu

**Affiliations:** 1 Institutes of Psychological Sciences, Hangzhou Normal University, Hangzhou, 311121 People’s Republic of China; 2 Center for Cognition and Brain Disorders, Hangzhou Normal University, Hangzhou, 311121 People’s Republic of China; 3 Zhejiang Key Laboratory for Research in Assessment of Cognitive Impairments, Hangzhou, 311121 People’s Republic of China; 4 Department of Psychology, Zhejiang Sci-Tech University, 5 Second Avenue, Xiasha Higher Education Zone, Hangzhou, 310018 People’s Republic of China

**Keywords:** Affective meaning, Conceptual meaning, Priming, Associative strength, LDT

## Abstract

To directly investigate the reciprocal causal relationship of the conceptual and affective meaning of words, two priming experiments were conducted with the lexical decision task. In Experiment 1, the influence of semantic relatedness on the affective priming effect was explored by manipulating the semantic associative strength between the prime and target words (i.e., high vs. low) while keeping the affective association between them constant (i.e., affectively congruent). In Experiment 2, the influence of the affective meaning on the semantic priming effect was explored by manipulating the emotional congruency of the prime and target words (i.e., congruent vs. incongruent) while keeping the semantic association between them constant (i.e., high associative strength). The results of Experiment 1 showed that when the semantic associative strength between the prime and target words was high, there was a significant affective priming effect, while no significant affective priming effect was found when the associative strength was low. The results of Experiment 2 revealed that in both the emotionally congruent and incongruent conditions, a significant semantic priming effect was obtained. These findings suggest that conceptual meaning is a more obligatory representation in words and that the processing of the affective meaning is constrained by the conceptual meaning of words.

## Introduction

As the basic unit of language, words are central to the comprehension of the language system. A word could convey both descriptive and evaluative information (Bühler 1990; Clore and Ketelaar 1997; Kövecses 2003). For example, the word ‘elephant’ refers to a large animal in most contexts, but it could also be used to depict something strong (i.e., ‘he looks like an elephant’); the word ‘happy’ mainly means cheerful and delighted, but it also refers to a type of emotion. In particular, a certain type of emotional words called dual-meaning words explicitly contain both descriptive and evaluative meaning (e.g., the dual-meaning word ‘oppressor’ conveys both the descriptive meaning of ‘emperor’ and the evaluative meaning of ‘cruel’) (Liu et al. 2010). Furthermore, our previous study demonstrated that segregated correlates and shared neural bases underlie the conceptual meaning and affective meaning of words (Liu et al. 2010).

Many researchers have explored the temporal relationship between the processing of descriptive and evaluative information in words. It has been argued that affective evaluation is the very first stage in object processing, which is termed ‘the affective primacy hypothesis’ (Zajonc 1980, 2000). According to this hypothesis, the retrieval of the evaluative or affective meaning precedes the retrieval of the descriptive or conceptual meaning of a word. Many studies adopting the priming paradigm, which is utilized to measure the affective or semantic association between the prime and target (Klauer and Musch 2003), have supported this claim. Studies have shown that reliable affective priming effects, whereby primes facilitate the encoding of affectively congruent targets, have been consistently observed at short stimulus onset asynchronies (SOAs) (less than 300 ms) (e.g., De Houwer et al. 2002; Fazio et al. 1986; Hermans et al. 1994, 2001; for a review, see Klauer and Musch 2003). These studies demonstrated that affective processing is more of an automatic process and is faster than semantic processing (De Houwer et al. 2002; Hermans et al. 1994, 2001; Murphy and Zajone 1993).

However, semantic processes could also be automatic (Neely 1991). Many studies adopting the semantic priming paradigm found that the semantic priming effect could occur not only at long SOAs (e.g., 1000 ms in Sperber et al. 1979; 1200 ms in Yap et al. 2016) but also at short SOAs (e.g., 35 ms in Burgess and Simpson 1988; 43 ms in Naccache and Dehaene 2001; 50 ms in Liu et al. 2010). Furthermore, some priming studies that adopted a comparative approach to compare the two types of priming effects (e.g., De Houwer et al. 2002; Ferré and Sánchez-Casas 2014; Klinger et al. 2000; Liu et al. 2010; Storbeck and Robinson 2004) found that significant semantic and affective priming effects could be observed at the same time at both short SOAs (e.g., 50 ms in Klinger et al. 2000 and Liu et al. 2010) and long SOAs (e.g., 250 ms in De Houwer et al. 2002).

The above mentioned studies all concerned the temporal relationship of the affective meaning and conceptual meaning of a word, i.e., conceptual processing precedes affective evaluation or vice versa. To our knowledge, no study to date has directly investigated the reciprocal causal relationship of the conceptual and affective meaning of a word. Several comparative studies addressing the relationship of semantic and affective priming, in which the semantic and affective relations between primes and targets were systematically manipulated, have provided some clues on the conceptual and affective meaning of a word. In a study by Hermans et al. (2002), significant affective priming effects were observed in an evaluative categorization task (Experiment 1, Experiment 2) and a LDT task (Experiment 3), even when the prime and target word pairs were associatively unrelated (Experiment 1, 2 and 3). These findings suggested that the affective meaning of a word may be more obligatory at encoding. However, in a study conducted by Storbeck and Robinson (2004), significant affective priming only appeared when the prime and target words came from a single semantic category, while semantic priming effects reliably emerged across different tasks (LDT and evaluation). These findings indicated that the affective meaning may be restricted by the conceptual meaning of a word. Moreover, a recent study (Ihmels et al. 2016) disentangled the emotional congruity and integrativity (which refers to the easy of integrating two concepts into a new meaningful compound representation), and found that evaluative-priming effects were confined to integrative prime–target pairs. In sum, although these studies concerning the relationship of the semantic and affective priming provided indirect evidences on the reciprocal relationship of the conceptual and affective meaning of a word, the findings were conflicting and could not provide a clear answer to the question. Thus, the present study attempted to address this issue directly. We aim to reveal whether the conceptual meaning could constrict the affective meaning of a word, or vice versa.

In the present study, two experiments were conducted using the priming paradigm, which is recommended as a promising method of exploring the relationship between different word meanings (e.g., dominant meaning (e.g., new–novel) and subordinate meaning (e.g., book–novel) of a word) (Faust and Lavidor 2003). By using the priming paradigm, investigators can explore the nature of the semantic information of a word by varying the prime–target relationships (Moss et al. 1995). Thus, the present Experiment 1 and Experiment 2 were designed in a systematical way. Experiment 1 tested whether the conceptual meaning could affect the affective meaning of a word. Experiment 2 explored whether the affective meaning could affect the conceptual meaning of a word. To this end, in Experiment 1, the prime and target words were kept affectively congruent, while the semantic relatedness between the prime and target was manipulated to be with high or low associative strength; in Experiment 2, the semantic relatedness between the prime and target was kept consistent (with high associative strength), while the affective congruency between the prime and target words was manipulated to be congruent or incongruent.

In this study, we used a short SOA (50 ms) in the priming paradigm, since affective priming is more of an automatic process (De Houwer et al. 2002; Hermans et al. 1994, 2001; Murphy and Zajone 1993). Following the comparative studies (e.g., Ferré and Sánchez-Casas 2014; Liu et al. 2010; Storbeck and Robinson 2004), the LDT task (in which participants must decide whether the target item is a real word) was used in the study since it does not explicitly favor either semantic or affective priming from a response competition perspective (De Houwer 2003).

## Experiment 1

In this experiment, we tested whether the conceptual meaning could affect the affective meaning of a word. Thus, we manipulated the extent of the semantic relatedness between the prime and target while keeping the affective association between them constant (i.e., affectively congruent). The associative strength between the prime and target words was used to measure the extent of the associative relations in semantic memory. Specifically, it was tested whether high and low associative strength would affect the affective priming effect in the experiment. In addition, two distinct irrelevant control conditions were utilized in the experiment that were associated separately with the high associative strength and the low associative strength condition. In such a design, the related condition and the unrelated or control condition could share the same target word. We predicted that only when the associative strength between the prime and target word was high would the affective priming effect occur.

## Method

### Participants

A total of 36 college students (23 females, mean age of 20.3 years with a range of 17–24 years) participated in this experiment for monetary compensation. All were right-handed native Mandarin Chinese speakers with normal or corrected-to-normal vision. Written informed consent was obtained from each participant in accordance with the guidelines and approval of the Institutional Review Board of the Department of Psychology of Zhejiang Sci-Tech University. All experimental methods were conducted in accordance with the approved guidelines regarding all the relevant aspects, including the recruitment, experimental process information, compensation and debriefing of participants.

### Materials and Design

The experiment adopted a typical affective priming paradigm with the LDT. The target word was either a dual-meaning emotional word (negative or positive) or a nonword. Emotional target words were preceded by a prime word that was either related or unrelated to the target. For the related condition, the associative strength of the prime and target semantics was deliberately manipulated, i.e., half of the pairs were strongly semantically related, and the other half were weakly semantically related. For the control condition, the prime word was neutral and unrelated to the target. Thus, the experiment consisted of four types of word pairs, i.e., highly semantically related primes and targets (termed HP) and the corresponding semantically unrelated control primes and targets (termed HC), lowly semantically related primes and targets (termed LP) and the corresponding semantically unrelated control primes and targets (termed LC). Table 1 shows examples of the stimuli used in Experiment 1.

**Table 1:** Material Examples of Experiment 1 and Experiment 2

Condition	Priming	
	Related	Unrelated
*Experiment 1*		
High associative strength	幸福–新娘 (happiness–bride)	环境–新娘 (environment–bride)
Low associative strength	残破–豺狼 (shabby–wolf)	柏油–豺狼 (tar–wolf)
*Experiment 2*		
Emotionally congruent	漂亮–美丽 (beautiful–pretty)	特殊–美丽 (special–pretty)
Emotionally incongruent	懦弱–勇敢 (cowardly–brave)	附近–勇敢 (nearby–brave)

Twenty positive and 20 negative words were used as target real words. A total of 40 positive, 40 negative and 80 neutral words were adopted as prime words to pair with the real target words. The emotional valence of the stimulus words was rated by 20 additional participants (who were not participants of the formal experiment) based on a 9-point Likert scale (1 = extremely negative, 9 = extremely positive).

Of the emotional prime words, half (20 positive and 20 negative words, termed P1) were strongly related to the target words, and the other half (termed P2) were weakly related to the target words. It should be noted that to strictly manipulate the strong and weak association, each word in the list of target words was paired with a certain prime word one at a time, which was either strongly or weakly semantically associated with the target word (but not with other target words). For each word in P1 and P2, there was a specific control neutral word unrelated to the corresponding target word. Therefore, there were two lists of control prime words (controls for P1 were termed C1 and those for P2 were termed C2; each control list consisted of 40 neutral words). There was no significant difference between the emotional and control prime words in the frequency of words (P1: 3866.6 ± 7458.3 per million (mean ± SD), C1: 3645.1 ± 6714.7 per million, *p* = 0.89; P2: 2724.9 ± 3713.2 per million, C2: 2774.0 ± 3236.9 per million, *p* = 0.95) (Modern Chinese Frequency Dictionary 1986) and the number of strokes of the characters constituting the words (P1: 16.5 ± 3.9, C1: 16.7 ± 4.4, *p* = 0.85; P2: 18.2 ± 3.1, C2: 17.8 ± 3.8, *p* = 0.61). There was no significant difference in the emotional valence between the two halves of prime words (for positive target words: P1: 7.6 ± 0.5, P2: 7.5 ± 0.3, *p* = 0.57; for negative target words: P1: 2.3 ± 0.7, P2: 2.6 ± 0.4, *p* = 0.20; for neutral control words: C1: 5.1 ± 0.1, C2: 5.0 ± 0.1, *p* = 0.38). There were significant differences between the emotional and control prime words in terms of the emotional valence (all *p*s < 0.001). The target words were also evenly divided into two parts (termed T1 and T2), which did not differ in emotional valence (positive words: T1: 7.4 ± 0.5, T2: 7.2 ± 0.7, *p* = 0.46; negative words: T1: 2.3 ± 0.4, T2: 2.6 ± 0.4, *p* = 0.17), word frequency (T1: 1201.8 ± 1397.6 per million, T2: 1625.1 ± 2289.4 per million, *p* = 0.49) or number of strokes (T1: 19.4 ± 5.5, T2: 19.4 ± 4.3, *p* = 1.00).

The associative strength between the prime and target words was rated by 20 additional participants (who were not participants of the formal experiment) based on a 9-point Likert scale (1 = not at all semantically associated, 9 = strongly semantically associated). The mean (± SD) associative strength of the prime and target pairs was 7.5 (± 1.3) for strongly related pairs (i.e., prime words in P1 and target words), 2.7 (± 1.5) for weakly related pairs (i.e., prime words in P2 and target words), 2.1 (± 1.5) for control pairs in the strong associative condition (i.e., prime words in C1 and target words) and 2.2 (± 1.6) for control pairs in the weak associative condition (i.e., prime words in C2 and target words). There were significant differences in associative strength between the strongly and weakly related pairs (*p *< 0.001), between strongly related pairs and the corresponding control pairs (*p *< 0.001), and between weakly related pairs and the corresponding control pairs (*p *< 0.01). There was no significant difference in the associative strength between the two types of control pairs (*p* = 0.18).

To control the repetition time of target words, the pairing of the prime and target words adopted the crossover design. For half of the participants, half of the target words (T1) were paired with the corresponding half of the prime words in P1 (to form the strongly associative condition) and with the corresponding half of the control words in C1 (to form the control condition corresponding to the strongly associative condition). Meanwhile, the other half of the target words (T2) were paired with the corresponding half of the prime words in P2 (to form the weakly associative condition) and with the corresponding half of the control words in C2 (to form the control condition corresponding to the weakly associative condition). For the other half of the participants, half of the target words (T2) were paired with the other half of the prime words in P1 (to form the strongly associative condition) and with the other half of the control words in C1 (to form the control condition corresponding to the strongly associative condition). Meanwhile, the other half of the target words (T1) were paired with the other half of the prime words in P2 (to form the weakly associative condition) and with the other half of the control words in C2 (to form the control condition corresponding to the weakly associative condition). Note that for each participant, only half of the total prime words were used.

Each participant had to complete 80 real word pairs in which a certain target word appeared twice (once with a strongly or weakly related prime word in P1 or P2 and once with a prime word in C1 or C2) in two separate sessions. No prime words were replicated for each participant. Of the strongly or weakly related pairs, the prime word and target word in each pair was emotionally congruent (i.e., both were positive or negative words).

For the sake of the LDT, there were also 80 nonwords as targets. The 40 real target words and 80 nonword targets did not differ significantly in the number of strokes (*p* = 0.91). For each nonword target, there was a prime word. To match with the real word pairs, another two types of prime words were paired with the nonword target, i.e., 40 emotional prime words and 40 neutral prime words. These prime words did not differ significantly from the corresponding prime words in the real word pairs in emotional valence (all *p*s > 0.5), word frequency (all *p*s > 0.1) or number of strokes (all *p*s > 0.1).

### Procedure

The experiment was conducted in a lightproof and soundproof room. A Windows PC controlled the stimulus presentation and response recording using the E-Prime 1.1 software with millisecond timing accuracy. Participants were seated in front of the computer at a distance of approximately 60 cm. All prompts and words were shown at the center of the screen, and the participants used the keyboard to respond. For each trial, the whole sequence was as follows: warning signal “+” (150 ms)—blank (500 ms)—prime word (50 ms)—target (≤ 2000 ms). The participants were instructed to judge whether the target was a real word or a nonword as quickly and accurately as possible. They pressed ‘A’ for a real word and ‘L’ for a nonword (the two response hands were counterbalanced across participants). If a participant responded within 2000 ms, the target would disappear immediately. The next trial started after the participant’s response or after the given time had elapsed. Participants were exposed to a short practice session before the formal sessions, and there was a short break between the two formal sessions.

## Results

Incorrect trials (3.87%) were excluded from the reaction time (RT) analyses, along with outlier trials in which the response latencies were beyond three standard deviations (0.78%) from the mean.

**Fig. 1:**
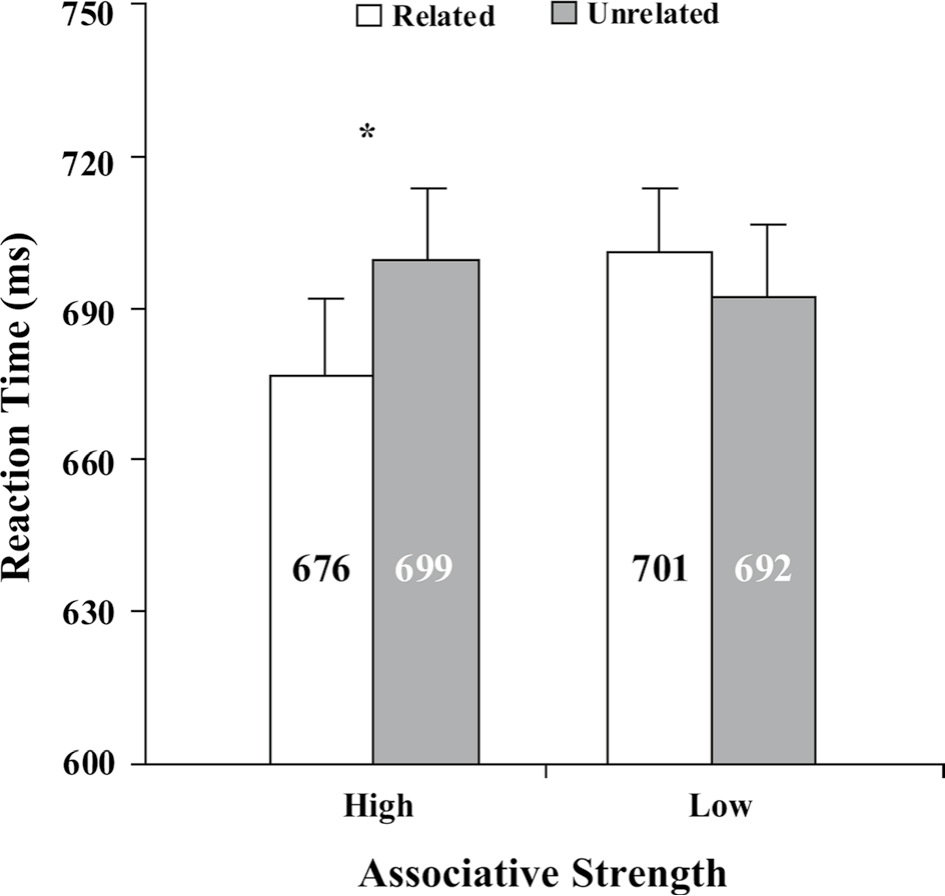
Mean reaction times for the related and unrelated conditions as a function of semantic associative strength (high, low) between the prime and target words. Error bars show standard errors. *p < 0.05

A paired *t* test revealed significant difference between the mean RTs in the HP trials and those in the HC trials [*t* (35) = − 2.54, *p* = 0.02, *d* = 0.42]. A paired t-test yielded no significant difference between the mean RTs in the LP trials and those in the LC trials [*t* (35) = 1.42, *p* = 0.17]. A paired t-test showed a significant difference between the two priming effects (i.e., (HC-HP) vs. (LC-LP)) [*t* (35) = 3.34, *p* < 0.005, *d* = 0.56]. See Fig. 1.

A paired t-test revealed no significant difference between the mean error rates in the HP trials and those in the HC trials [*t* (35) = − .37, *p* = 0.71], and no significant difference between the mean error rates in the LP trials and those in the LC trials [*t* (35) = 1.03, *p* = 0.31].

The results of Experiment 1 showed that when the semantic associative strength between the prime and target words was high, there was a significant affective priming effect; however, when the associative strength was low, there was no significant affective priming effect. These results were consistent with our prediction, meaning that the semantic associative strength between the prime and target word could influence the affective priming effect. Thus, the results of Experiment 1 suggested that the conceptual meaning of a word could affect the affective meaning.

## Experiment 2

In this experiment, we tested whether the affective meaning could affect the conceptual meaning of a word. Thus, we manipulated the emotional congruency of the prime and target words while keeping the semantic association between them constant. Based on the results of Experiment 1, we chose to use prime-target pairs with high associative strength. Specifically, we tested whether emotional congruency affected the semantic priming effect in the experiment. In this experiment, two distinct irrelevant control conditions were also adopted that were separately associated with the affectively congruent and incongruent conditions. We predicted that there would be a significant semantic priming effect in the emotionally congruent but not in the incongruent condition.

## Method

### Participants

A total of 36 college students (20 females, mean age of 20.5 years with a range of 17–24 years) participated in this experiment. The recruitment criteria and procedure were identical to those of Experiment 1.

### Materials and Design

The experiment also used the priming paradigm with the LDT. The target word was either an emotional word (negative or positive) or a nonword. Emotional target words were preceded by a prime word that was either related or unrelated to the target. For the related condition, the associative strength of the prime and target semantics was kept very high. However, the emotional congruency of the prime and target words was deliberately manipulated, i.e., half of the pairs were emotionally congruent, and the other half of the pairs were emotionally incongruent. For the control condition, the prime word was neutral and unrelated to the target. Thus, the experiment consisted of four types of word pairs, i.e., emotionally congruent related primes and targets (termed CP) and the corresponding unrelated control primes and targets (termed CC), emotionally incongruent related primes and targets (termed IP) and the corresponding unrelated control primes and targets (termed IC). Table 1 shows examples of the stimuli used in Experiment 2.

Twenty positive and 20 negative words were used as target real words. A total of 40 positive, 40 negative and 80 neutral words were adopted as prime words to pair with the real target words. The rating of the emotional valence of the stimuli was identical to that in Experiment 1.

Of the emotional prime words, half (20 positive and 20 negative words, termed P1) were emotionally congruent with the target words, and the other half (termed P2) were emotionally incongruent with the target words. There were two lists of control prime words (controls for P1 were termed C1 and those for P2 were termed C2; each control list consisted of 40 neutral words). There were no significant differences between the emotional and control prime words in word frequency (P1: 2987.6 ± 5479.7 per million, C1: 2278.9 ± 2265.4 per million, *p* = 0.61; P2: 3389.4 ± 6367.7 per million, C2: 3635.7 ± 5079.1 per million, *p* = 0.89) or number of strokes (P1: 17.7 ± 4.5, C1: 17.7 ± 4.0, *p* = 1.00; P2: 18.6 ± 6.2, C2: 18.0 ± 4.1, *p* = 0.72). There was significant difference in emotional valence between the two halves of the emotional prime words (for positive target words: P1: 7.5 ± 0.3, P2: 3.0 ± 0.4, *p* < 0.001; for negative target words: P1: 2.5 ± 0.2, P2: 7.4 ± 0.5, *p* < 0.001) while no significance occurred in emotional valence between the two neutral controls (C1: 5.1 ± 0.2, C2: 5.1 ± 0.2, *p* = 0.76). There were significant differences between the emotional and control prime words in terms of emotional valence (all *p*s < 0.01). The target words were also divided into two equal parts, which did not differ in emotional valence (positive words: T1: 7.4 ± 0.2, T2: 7.2 ± 0.5, *p* = 0.45; negative words: T1: 2.5 ± 0.4, T2: 2.5 ± 0.5, *p* = 0.95), word frequency (T1: 2823.1 ± 3961.0 per million, T2: 4849.4 ± 8684.3 per million, *p* = 0.36) or number of strokes (T1: 19.2 ± 4.2, T2: 18.8 ± 4.5, *p* = 0.80).

The associative strength between the prime and target words was rated by seventeen additional participants (who were not participants of the formal experiment) with a procedure identical to that of Experiment 1. The mean (± SD) associative strength was 7.7 (± 1.7) in the emotionally congruent pairs and 7.3 (± 1.7) in the emotionally incongruent pairs, and there was no significant difference between the two conditions (*p* = 0.11). In addition, the above two types of associative strength were both significantly greater than 5 (*p*s < 0.001), indicating that the prime and target words in both the congruent and incongruent pairs were strongly semantically associated.

To control the repetition time of the target words, the pairing of the prime and target words adopted the crossover design. The pairing principle and method were identical to those of Experiment 1. Each participant had to complete 80 real word pairs in which a certain target word appeared twice (once with an emotionally congruent or incongruent related prime word in P1 or P2 and once with a prime word in C1 or C2) in two separate sessions. No prime words were replicated for each participant.

There were also 80 nonwords as targets paired with another 40 emotional prime words and 40 neutral prime words. These prime words did not differ significantly from the corresponding prime words in the real word pairs in emotional valence (all *p*s > 0.5), word frequency (all *p*s > 0.5) or number of strokes (all *p*s > 0.5).

### Procedure

The procedure of Experiment 2 was identical to that of Experiment 1.

## Results

Incorrect trials (3.26%) were excluded from the RT analyses along with outlier trials in which the response latencies were beyond three standard deviations (0.6%) from the mean.

**Fig. 2:**
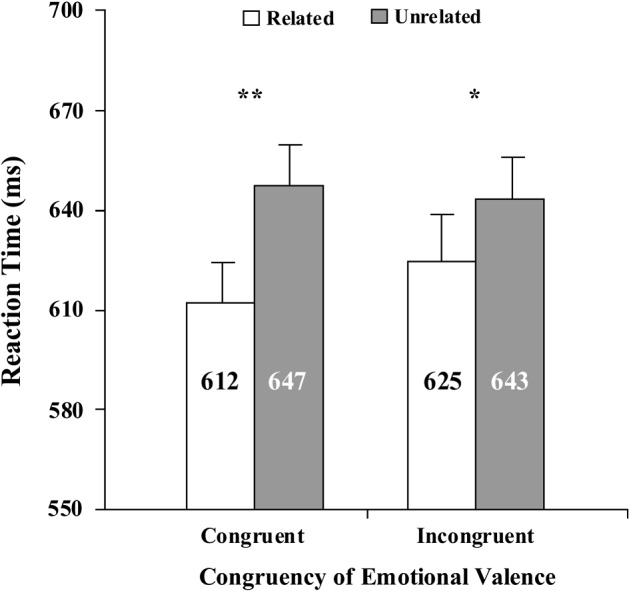
Mean reaction times for the related and unrelated conditions as a function of emotional congruency (congruent, incongruent) of the prime and target words. Error bars show standard errors. *p < 0.05, **p < 0.01

A paired t-test revealed significant difference between the mean RTs in the CP condition and those in the CC condition [*t* (35) = − 6.87, *p* < 0.001, *d* = 1.14], and significant difference between the mean RTs in the IP condition and those in the IC condition [*t* (35) = − 2.42, *p* = 0.02, *d* = 0.40]. A paired t-test showed marginally significant difference between the two priming effects (i.e., (CC-CP) vs. (IC-IP)) [*t* (35) = 1.88, *p* = 0.068]. See Fig. 2.

A paired t-test was also conducted on error rates. There was no significant difference between the mean error rates in the CP condition and those in the CC condition [*t* (35) = -1.44, *p* = 0.16], and no significant difference between the mean error rates in the IP condition and those in the IC condition [*t* (35) = 0.98, *p* = 0.33].

The results of Experiment 2 showed that when the semantic associative strength between prime and target words was high, there was a significant semantic priming effect whether the emotional valence of the prime and target was congruent or incongruent. These results were partly in agreement with our prediction. It was expected that a significant semantic priming effect would occur in the affectively congruent but not in the incongruent condition. However, the significance also appeared in the incongruent trials. Thus, when the semantic associative strength between the prime and target word was high, affective congruency could not modulate the semantic priming effect. Thus, the results of Experiment 2 suggested that the affective meaning could not affect the conceptual meaning of a word.

## General Discussion

The current study is the first attempt to directly address the causal relations of the conceptual meaning and affective meaning of words. In this study, two priming experiments were conducted to systematically investigate the reciprocal influence of the conceptual meaning and affective meaning of words. Experiment 1 explored the influence of semantic relatedness on the affective priming effect, and the results showed that when the semantic associative strength between the prime and target word was high, there was a significant affective priming effect, while no significant affective priming effect was found when the associative strength was low. Experiment 2 explored the influence of the affective meaning on the semantic priming effect, and the results showed that in both the emotionally congruent and incongruent conditions, a significant semantic priming effect was obtained in the context of a strong semantic association between the prime and target words. In total, these findings support the view that the conceptual meaning can constrain the affective meaning of a word.

Experiment 1 found a significant affective priming effect only when the associative strength between the prime and target words was high. When the prime and target words were highly semantically related, the priming effect may conflate both the affective and semantic priming, when comparing with the control condition in which the prime-target pairs were affectively and semantically unrelated. Since no significant affective priming effect was found when the associative strength between the prime and target words was low, in which the prime and target words were still affectively congruent, the significant priming effect in the high associative strength condition was essentially a semantic priming effect. The present results were consistent with those of Storbeck and Robinson (2004). In their study, both the semantic and affective relations between primes and targets were orthogonally manipulated, and the semantic and affective priming was systematically compared. They found a significant semantic priming effect in Study 1 (LDT task) and Study 2 (evaluation task), and the affective priming effect could occur only when the prime and target words came from a single semantic category in Study 3 (evaluation task). Their findings suggested that encoding semantic rather than affective meaning of objects was more obligatory. Several other studies were also in accordance with our results. For example, Kemp-Wheeler and Hill (1992) found a semantic but not an affective priming effect in a pronunciation task. De Houwer et al. (1998) obtained similar results using the pronunciation task. As the pronunciation task is a relatively pure index of semantic activation (Balota and Lorch 1986), these findings suggested that the affective meaning may be parasitic on the semantic meaning. The nonsignificant affective priming effect in the low associative strength condition was consistent with the findings of Ihmels et al. (2016). They found that the processing facilitation of prime-congruent targets could only appear when the prime and target words could be integrated into a meaningful concept. In the present Experiment 1, though the low associative prime-target pairs share the same emotional valence (e.g., shabby–wolf), but they could not be integrated semantically, thus could not lead to significant priming effect.

However, the absence of an affective priming effect in the low associative strength condition seems to be inconsistent with the existing affective priming studies. Affective priming is a widespread phenomenon at short SOAs (e.g., De Houwer et al. 2002; Fazio et al. 1986; Hermans et al. 2001; for a review, see Klauer and Musch 2003) and is regarded as an automatic process (De Houwer et al. 2002; Hermans et al. 1994, 2001; Murphy and Zajone 1993). However, the automatic processing of the affective information of a word does not mean that it is processed unconditionally (Lazarus 1999). In typical affective priming studies, though the semantic association between prime and target stimuli (words, pictures, etc.) was not controlled, it was always the case that the semantic association between primes and targets was relatively strong (e.g., happy–bride), thus resulting in a significant affective priming effect (Ferré and Sánchez-Casas 2014; Hermans et al. 2002). When the category (e.g., Storbeck and Robinson 2004), the semantic associative strength (e.g., the present study) or the relational integrativity (e.g., Ihmels et al. 2016) was deliberately manipulated, the affective priming effect was obtained only when the prime and target word pairs were from the same semantic category or were strongly semantically associated or integrative. In Experiment 1, the absence of the affective priming effect in the ‘low associative strength’ condition may be due to the reduced activation of the corresponding semantic representations. However, the absence of the affective priming effect in the ‘low associative strength’ condition in Experiment 1 seemed to be inconsistent with the results of Ihmels et al. (2016); they found a significant affective priming effect in a LDT task when the prime and target word pairs were associatively unrelated but not when they are related (Experiment 3). The inconsistency may be due to the methodological difference. In Ihmels et al.’s (2016) study, the affective priming was obtained from the comparison of affective congruent versus incongruent trials, while in the current study, the affective priming effect was calculated by comparing the emotional congruent trials with the neutral-prime control trials. As for the absence of affective priming effect for associatively related prime-target pairs, Ihmels et al. (2016) contributed it to the floor-effect, as the overall reaction times for the related trials were so fast (mean = 463 ms) after further speeding up by the associative context.

Experiment 2 showed that when the semantic associative strength was high, there was a significant semantic priming effect in both the emotionally congruent and incongruent condition. In a typical affective priming paradigm, the priming effect was measured by subtracting the RT of the emotionally congruent condition from that of the emotionally incongruent condition. In some studies that used neutral or unrelated controls, the emotionally incongruent condition was found to induce interference effect of target processing (Fazio et al. 1986; for reviews see De Houwer et al. 2009; Klauer and Musch 2003). However, in our Experiment 2, the emotionally incongruent primes also facilitated the processing of the semantically related target words. Thus, the significant priming effect in such a situation could not be due to the affective priming effect. In the emotionally incongruent condition, the semantic associative strength between the prime and target word was high, meaning that the prime and target also had a close semantic relationship. This relationship can promote activation of the other side, thus contributing to the target processing (Klauer et al. 2009) and triggering a significant semantic priming effect. Our results were also consistent with the findings of Ihmels et al. (2016). When the prime and target words were highly semantically associative, they could probably be integrated into a new representation (e.g., beautiful–pretty, cowardly–brave), thus resulting in significant priming effect.

In Experiment 1, the priming and target word have the same emotional valence in the related condition. According to the spreading activation theory (Collins and Loftus 1975), when the prime word was presented, the emotional information of the prime word was activated, which would lead to the facilitation of emotional information processing of the target word and thus trigger the affective priming effect. Here, the affective priming effect is a means of detecting the affective meaning processing of words. Our results revealed that the affective priming effect occurred only when the semantic associative strength was high. This result indicated that the affective meaning of a word could be constricted by the conceptual meaning. In Experiment 2, the prime and target word were strongly semantically associated (i.e., with high semantic associative strength) in the related condition. When the prime word was presented, the conceptual information of the prime word was activated, which would lead to the facilitation of the conceptual information processing of the target word, and thus trigger the semantic priming effect. Here, the semantic priming effect is a means of detecting the conceptual meaning processing of words. We found significant semantic priming effects regardless of whether the prime and target words were emotionally congruent or incongruent, which suggested that the conceptual meaning of a word could not be constrained by the affective meaning. Taken together, our findings suggested that the conceptual meaning may be a more robust representation than the affective meaning of a word.

Although both conceptual and affective features are represented in a semantic network (De Houwer and Hermans 1994), they are not exactly equal in the semantic representations of words. Our study found that the conceptual meaning is a more obligatory representation in words. The reason may be that affective priming is not based on associative connections within long-term memory (Klauer et al. 1997), whereas semantic priming is (Neely 1991); thus, conceptual analysis is more obligatory at encoding (Storbeck and Robinson 2004). Our results favored the cognitive primacy hypothesis (Lazarus 1984). It has been suggested that semantic processing is the precondition for information processing in affective routes (Rolls 1999), and people can identify how they feel about a stimulus only after they know what it is (Clore and Ketelaar 1997). A number of physiological studies also provided evidences for this hypothesis. It was found that the amygdala relies heavily on information from the inferior temporal cortex (area IT) to respond to affective stimuli (Fukuda et al. 1987; Kreiman et al. 2002; for a review, see Storbeck et al. 2006). Since area IT is part of the visual cortex and is associated with categorizing visual stimuli, these results favor the idea that semantic processing precedes affective retrieval in visual processing (Storbeck et al. 2006). Nevertheless, our findings need further confirmation (e.g., adopting different paradigms other than the LDT).

In sum, the current study directly addressed the causal relationship between the conceptual meaning and affective meaning of words using the LDT. Our results suggest that the conceptual meaning is a more obligatory representation in words and that the processing of the affective meaning may be dependent on and is constrained by the conceptual meaning of a word.

## References

[CR1] Balota D, Lorch R (1986). Depth of automatic spreading activation: Mediated priming effects in pronunciation but not in lexical decision. Journal of Experimental Psychology. Learning, Memory, and Cognition.

[CR2] Beijing Language College Language Instruction Institute (1986). Modern chinese frequency dictionary (in Chinese).

[CR3] Bühler K (1990). Theory of language: The representational function of language.

[CR4] Burgess C, Simpson GB (1988). Cerebral hemispheric mechanisms in the retrieval of ambiguous word meanings. Brain Language.

[CR5] Clore GL, Ketelaar T, Wyer RS (1997). Minding our emotions: On the role of automatic, unconscious affect. Advances in social cognition.

[CR6] Collins AM, Loftus EF (1975). A spreading-activation theory of semantic processing. Psychological Review.

[CR7] De Houwer J, Musch J, Klauer KC (2003). A structural analysis of indirect measures of attitudes. The psychology of evaluation: Affective processes in cognition and emotion.

[CR8] De Houwer J, Hermans D (1994). Differences in the affective processing of words and pictures. Cognition and Emotion.

[CR9] De Houwer J, Hermans D, Eelen P (1998). Affective and identity priming with episodically associated stimuli. Cognition and Emotion.

[CR10] De Houwer J, Hermans D, Rothermund K, Wentura D (2002). Affective priming of semantic categorisation responses. Cognition and Emotion.

[CR11] De Houwer J, Teige-Mocigemba S, Spruyt A, Moors A (2009). Implicit measures: a normative analysis and review. Psychological Bulletin.

[CR100] Faust M, Lavidor M (2003). Semantically convergent and semantically divergent priming in the cerebral hemispheres: Lexical decision and semantic judgment. Cognitive Brain Research.

[CR12] Fazio RH, Sanbonmatsu DM, Powell MC, Kardes FR (1986). On the automatic activation of attitudes. Journal of Personality and Social Psychology.

[CR13] Ferré P, Sánchez-Casas R (2014). Affective priming in a lexical decision task: is there an effect of words’ concreteness?. Psicológica.

[CR14] Fukuda M, Ono T, Nakamura K (1987). Functional relations among infero-temporal cortex, amygdala, and lateral hypothalamus in monkey operant feeding behavior. Journal of Neurophysiology.

[CR15] Hermans D, De Houwer J, Eelen P (1994). The affective priming effect: Automatic activation of evaluative information in memory. Cognition and Emotion.

[CR16] Hermans D, De Houwer J, Eelen P (2001). A time course analysis of the affective priming effect. Cognition and Emotion.

[CR17] Hermans D, Smeesters D, De Houwer J, Eelen P (2002). Affective priming for associatively unrelated primes and targets. Psychologica Belgica.

[CR18] Ihmels M, Freytag P, Fiedler K, Alexopoulos T (2016). Relational integrativity of prime-target pairs moderates congruity effects in evaluative priming. Memory & Cognition.

[CR19] Kemp-Wheeler SM, Hill AB (1992). Semantic and emotional priming below objective detection threshold. Cognition and Emotion.

[CR20] Klauer K, Musch J, Musch J, Klauer KC (2003). Affective priming: Findings and theories. The psychology of evaluation: Affective processes in cognition and emotion.

[CR21] Klauer K, Roßnagel C, Musch J (1997). List-context effects inevaluative priming. Journal of Experimental Psychology. Learning, Memory, and Cognition.

[CR22] Klauer KC, Teige-Mocigemba S, Spruyt A (2009). Contrast effects in spontaneous evaluations: A psychophysical account. Journal of Personality and Social Psychology.

[CR23] Klinger M, Burton P, Pitts S (2000). Mechanisms of unconscious priming: I. Response competition, not spreading activation. Journal of Experimental Psychology. Learning, Memory, and Cognition.

[CR24] Kövecses Z (2003). Metaphor and emotion: Language, culture, and body in human feeling.

[CR25] Kreiman G, Fried I, Koch C (2002). Single neuron correlates of subjective vision in the human medial temporal lobe. Proceedings of the National Academy of Sciences.

[CR26] Lazarus RS (1984). On the primacy of cognition. American Psychologist.

[CR27] Lazarus RS, Dalgleish T, Power M (1999). The cognition-emotion debate: A bit of history. Handbook of cognition and emotion.

[CR28] Liu HL, Hu ZG, Peng DL, Yang YH, Li KC (2010). Common and segregated neural substrates for automatic conceptual and affective priming as revealed by event-related functional magnetic resonance imaging. Brain and Language.

[CR29] Moss HE, Ostrin RK, Tyler LK, Marslen-Wilson WD (1995). Accessing different types of lexical semantic information: Evidence from priming. Journal of Experimental Psychology. Learning, Memory, and Cognition.

[CR30] Murphy ST, Zajone RB (1993). Affect, cognition, and awareness: Affective priming with optimal and suboptimal stimulus exposures. Journal of Personality and Social Psychology.

[CR31] Naccache L, Dehaene S (2001). Unconscious semantic priming extends to novel unseen stimuli. Cognition.

[CR32] Neely JH, Besner D, Humphreys GW (1991). Semantic priming effects in visual word recognition: A selective review of current findings and theories. Basic processes in reading: Visual word recognition.

[CR200] Rolls ET (1999). The brain and emotion.

[CR33] Sperber RD, McCauley C, Ragain RD, Well CM (1979). Semantic priming effects on picture and word processing. Memory & Cognition.

[CR34] Storbeck J, Robinson MD (2004). Preferences and inferences in encoding visual objects: A systematic comparison of semantic and affective priming. Personality and Social Psychology Bulletin.

[CR35] Storbeck J, Robinson MD, McCourt ME (2006). Semantic processing precedes affect retrieval: The neurological case for cognitive primacy in visual processing. Review of General Psychology.

[CR36] Yap MJ, Hutchison KA, Tan LC, Jones MN (2016). Individual differences in semantic priming performance. Big data in cognitive science: From methods to insights.

[CR37] Zajonc RB (1980). Feeling and thinking: Preferences need no inferences. American Psychologist.

[CR38] Zajonc RB, Forgas JP (2000). Feeling and thinking: Closing the debate over the independence of affect. Feeling and thinking: The role of affect in social cognition.

